# Association of Pericardiac Adipose Tissue With Coronary Artery Disease

**DOI:** 10.3389/fendo.2021.724859

**Published:** 2021-09-06

**Authors:** Mingxuan Li, Lin Qi, Yanglei Li, Shuyi Zhang, Lei Lin, Lijin Zhou, Wanlin Han, Xinkai Qu, Junfeng Cai, Maoqing Ye, Kailei Shi

**Affiliations:** ^1^Department of Cardiology, Huadong Hospital Affiliated to Fudan University, Shanghai, China; ^2^Department of Computed Tomography, Huadong Hospital Affiliated to Fudan University, Shanghai, China; ^3^Department of Cardiovascular Surgery, Huadong Hospital Affiliated to Fudan University, Shanghai, China; ^4^Shanghai Key Laboratory of Clinical Geriatric Medicine, Huadong Hospital Affiliated to Fudan University, Shanghai, China

**Keywords:** CAD (Coronary artery disease), PAT (pericardial adipose tissue), inflammation, macrophage cell, bioinformation

## Abstract

**Background and Aim:**

Coronary artery disease (CAD) poses a worldwide health threat. Compelling evidence shows that pericardial adipose tissue (PAT), a brown-like adipose adjacent to the external surface of the pericardium, is associated with CAD. However, the specific molecular mechanisms of PAT in CAD are elusive. This study aims to characterize human PAT and explore its association with CAD.

**Methods:**

We acquired samples of PAT from 31 elective cardiac surgery patients (17 CAD patients and 14 controls). The transcriptome characteristics were assessed in 5 CAD patients and 4 controls *via* RNA-sequencing. Cluster profile R package, String database, Cytoscape were applied to analyze the potential pathways and PPI-network key to DEGS, whereas the hubgenes were predicted *via* Metascape, Cytohubba, and MCODE. We use Cibersort, ENCORI, and DGIDB to predict immunoinfiltration, mRNA-miRNA target gene network, and search potential drugs targeting key DEGs. The predictable hubgenes and infiltrating inflammatory cells were validated in 22 patients (12 CAD samples and 10 control samples) through RT-qPCR and immunohistochemistry.

**Results:**

A total of 147 different genes (104 up-regulated genes and 43 down-regulated genes) were identified in CAD patients. These different genes were associated with immunity and inflammatory dysfunction. Cibersort analysis showed monocytes and macrophages were the most common subsets in immune cells, whereas immunohistochemical results revealed there were more macrophages and higher proportion of M1 subtype cells in PAT of CAD patients. The PPI network and module analysis uncovered several crucial genes, defined as candidate genes, including Jun, ATF3, CXCR4, FOSB, CCl4, which were validated through RT-qPCR. The miRNA-mRNA network implicated hsa-miR-185-5p as diagnostic targets and drug-gene network showed colchicine, fenofibrate as potential therapeutic drugs, respectively.

**Conclusion:**

This study demonstrates that PAT is mainly associated with the occurrence of CAD following the dysfunction of immune and inflammatory processes. The identified hubgenes, predicted drugs and miRNAs are promising biomarkers and therapeutic targets for CAD.

## Introduction

Coronary artery disease (CAD) is a global health threat, particularly due to its high level of morbidity, which poses an enormous socioeconomic and medical burden (1). Obesity, a type of metabolic syndrome characterized by abnormal deposition of body fat with chronic inflammation of adipose tissue, is associated with multiple cardiovascular diseases, including CAD (2). According to the Framingham Heart Study, the risk factors for CAD are more associated with omental adipose tissue than subcutaneous adipose tissue (SAT) (3). These observations may be plausible owing to the differences in adipose tissue endocrine and metabolism. White adipose tissue (WAT) and brown adipose tissue (BAT) are the two major types of adipose tissues. Briefly, WAT comprises adipocytes with a large, single fat droplet and is presumed as the main depot for lipid storage, whereas BAT comprises several smaller fat droplets and numerous mitochondria and plays a role in heat production (4). In humans, BAT was thought to rapidly involute and essentially disappear within the first years after birth, only a small amount is found in the scapula, paraspinal, and around the heart and aorta in adulthood (5).

Pericardial adipose tissue (PAT), which refers to the fat surrounding the external surface of the pericardium, is supplied by the internal mammary artery (6). PAT covers the pericardium which is closely adjacent to the epicardial adipose tissue (EAT), coronary artery, and the heart. Scholars have suggested that PAT may play a vital role in cardiovascular disease (7–9). Previous reports in humans indicate that PAT may appear brown-like adipocyte in morphology, with distinct features different from WAT and BAT (10–12). Elsewhere, PAT, as a metabolically active endocrine local adipocyte depot, was found to be associated with coronary artery disease (CAD) through the production of free fatty acids and pro-and anti-inflammatory adipocytokines (13). Elevated PAT volume is known to be associated with coronary atherosclerosis, hypoadiponectinemia, inflammation and represents the highest risk factor for atherosclerosis (14). Other reports have further demonstrated the association of PAT with cardiovascular events and left ventricular remodeling (15, 16). However, whether the transcriptome of PAT changes during CAD, the molecular mechanism by which PAT mediates CAD progress, and the possibility to improve the function of PAT in CAD treatment remains elusive.

In the present study, we employed the RNA-sequencing (RNA-Seq) approach to explore the transcriptome characteristic shift in PAT from humans undergoing cardiac surgery with or without CAD. This was followed by the analysis of the functional enrichment, protein-protein interaction (PPI) network, hubgenes, and microRNA (miRNA)-mRNA regulatory network. We further predicted the potential drugs that target the key differentially expressed genes (DEGs). This study will deepen our understanding of PAT in the pathogenesis of CAD.

## Study Patients

### Subject Recruitment

This study complied with the Declaration of Helsinki and was approved by the ethics committee of Huadong Hospital Affiliated to Fudan University, Shanghai, China (2020K082). All patients signed written informed consent, and underwent preoperative coronary angiography. Control patients were referred for elective valve surgery and exhibited no significant CAD (a single lesion >50%) on preoperative coronary angiograms. Besides, CAD patients were referred for coronary artery bypass (CABG) surgery because of significant stenosis and surgical indications. Eventually, 31 patients, including 17 CAD patients undergoing coronary artery bypass grafting (CABG) and 14 control patients undergoing valve replacement or valve repair were enrolled for analysis.

### Sample Collection and Preparation

**Figure 1:**
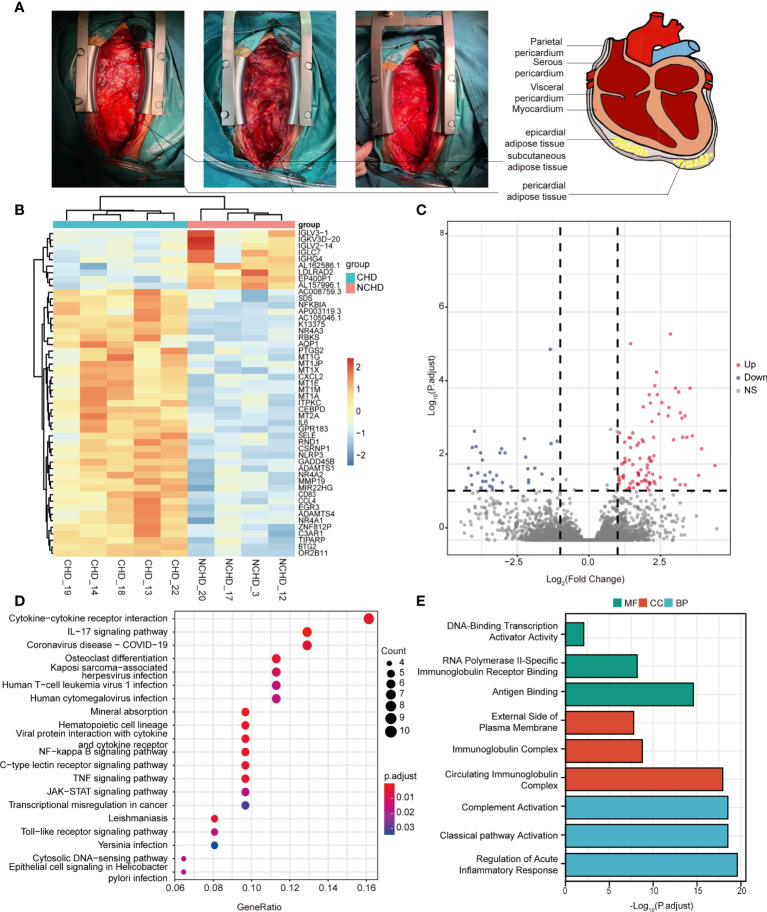
**(A)** Localization of pericardial fat; **(B)** Heatmap results of DEGs; **(C)** Volcano plot results of DEGs; **(D, E)** The significantly enriched KEGG and GO terms that correspond to coding gene functions of upregulated and downregulated DEGs.

We took a sample of PAT (1.0 g) adjacent to the pericardial surface during surgery (**Figure 1A**). The adipose sections collected from 5 CAD and 4 control patients were kept in 10% formalin for histological analysis. The other adipose tissues were immediately snap-frozen and stored at liquid nitrogen (–196°C). Five CAD and 4 control adipose tissue samples underwent RNA sequencing, while the rest were used in RT-PCR analyses.

### Histology and Immunohistochemistry

Formalin-fixed adipose tissues were embedded in paraffin and sectioned (8μm thick) and stained with hematoxylin and eosin (H&E). H&E-stained sections revealed the adipocyte size. To quantify M1 or M2 macrophages, cryosections were stained with rabbit anti-human CD11b (1:1000 dilution) (Abcam, UK) and rabbit anti-human CD68 (1:1000 dilution) (Abcam, UK) antibodies after which a goat anti-rabbit secondary antibody was conjugated to horseradish peroxidase. Immunohistochemical images were captured using a Zeiss LSM 5 Pascal microscope (M205FA, Zeiss, Oberkochen, Germany). Image-Pro Plus software (version6.0, MEDIA CYBERNETICS, USA) was employed to analyze the images.

### mRNA Isolation and Real-Time PCR

Total RNA was isolated from adipose tissue biopsies using an RNA rapid extraction kit (TR205-200, Tianmo biotech, Beijing, China) following the manufacturer’s protocol. RT-PCR was performed using a cDNA reverse transcription kit (RR047A, TAKARA, Japan), Premix Ex Taq (RR420A, TAKARA, Japan) in 7500 Real-Time PCR system (Applied Biosystems, San Francisco, CA, USA). Standard and melting curves were generated in every plate for each gene to ensure that the reaction is efficient and specific. The cycle threshold value of β-actin acted as the internal control. The relative expression levels of different genes were analyzed *via* the 2−ΔΔCT method. Primer sequences were obtained from PrimerBank (http://pga.mgh.harvard.edu/ primerbank) as follows (**Table 1**): JUN forward TCCAAGTGCCGAAAAAGGAAG, JUN reverse CGAGTTCTGAGCTTTCAAGGT; ATF3 forward CGAGTTCTGAGCTTTCAAGGT, ATF3 reverse TTCTTTCTCGTCGCCTCTTTTT; CXCR4 forward ACTACACCGAGGAAATGGGCT, CXCR4 reverse CCCACAATGCCAGTTAAGAAGA; FOSB forward GCTGCAAGATCCCCTACGAAG, FOSB reverse ACGAAGAAGTGTACGAAGGGTT; CCL4 forward CTGTGCTGATCCCAGTGAATC, CCL4 reverse TCAGTTCAGTTCCAGGTCATACA; CXCL2 forward CTCAAGAATGGGCAGAAAGC, CXCL2 reverse CTCCTAAGTGATGCTCAAAC (17).

**Table 1:** The primer sequences about hub genes.

Gene symbal	Forward Primer	Reverse Primer
JUN	TCCAAGTGCCGAAAAAGGAAG	CGAGTTCTGAGCTTTCAAGGT
ATF3	CGAGTTCTGAGCTTTCAAGGT	TTCTTTCTCGTCGCCTCTTTTT
CXCR4	ACTACACCGAGGAAATGGGCT	CCCACAATGCCAGTTAAGAAGA
FOSB	GCTGCAAGATCCCCTACGAAG	ACGAAGAAGTGTACGAAGGGTT
CCL4	CTGTGCTGATCCCAGTGAATC	TCAGTTCAGTTCCAGGTCATACA
CXCL2	CTCAAGAATGGGCAGAAAGC	CTCCTAAGTGATGCTCAAAC

### RNA Sequencing

Paired-end libraries were synthesized using the TruSeq™ RNA Sample Preparation Kit (Illumina, USA) following the TruSeq™ RNA sample preparation guide. Briefly, the poly-A containing mRNA molecules were purified using poly-T oligo-attached magnetic beads. Thereafter, the mRNA was fragmented into small pieces using divalent cations under 94°C for 8 min. The cleaved RNA fragments were copied into first-strand cDNA using reverse transcriptase and random primers. Subsequently, the second strand cDNA was synthesized using DNA Polymerase I and RNase H. These cDNA fragments then underwent an end repair process, the addition of a single ‘A’ base, and ligation of the adapters. The products were purified and enriched with PCR to generate the final cDNA library. Clean libraries were quantified using a Qubit^®^ 2.0 Fluorometer (Life Technologies, USA), and validated by Agilent 2100 bioanalyzer (Agilent Technologies, USA) to confirm the insert size and evaluate the mole concentration. Cluster was generated using cBot with the library diluted to 10 pM and then was sequenced on the Illumina NovaSeq 6000 (Illumina, USA). Library construction and sequencing were performed by Sinotech Genomics Co., Ltd (Shanghai, China).

### Data Processing of DEGs

Gene abundance was expressed as fragments per kilobase of exon per million reads mapped (FPKM). We employed the Stringtie software to count the fragments within each gene. TMM algorithm was applied for normalization. The DEGs between CAD and control patients were detected in the Illumina data collection software, whereby the P-value and |log_2_FC| were calculated. Genes that met the cutoff criteria, P value<0.05 and |log_2_FC|>1.0, considered as significantly modulated, were retained for subsequent analysis.

### Enrichment Analysis

The Gene Ontology (GO) analysis and a Kyoto Encyclopedia of Genes and Genomes (KEGG) pathways analysis were performed in the R package cluster Profiler (version 3.18.0) (18). GO enrichment analysis included biological processes (BP), cellular components (CC), and molecular functions (MF).

### PPI Network Construction, Significant Module, and Hub Genes Analysis

The PPI network was first analyzed using Cytoscape (version 3.7.2, www.cytoscape.org) software after which the key genes in the PPI networks were identified using cytohubba (version 1.4.2), a plug-in of the Cytoscape software (19, 20). The Molecular Complex Detection tool (MCODE) (version 1.5.1) and Metascape (http://metascape.org/gp/) were employed to screen the significant module (21, 22).

### Identification and Analysis of Significant Genes

A Venn diagram was delineated to identify significant common genes across “Metascape_MCODE”, “Cytoscape_MCODE”, and “Cytoscape_cytoHubba” by Veeny2.1 (https://bioinfogp.cnb.csic.es/tools/venny/). Summarized functions of significant genes were obtained *via* GeneCards (https://www.genecards.org/) (23).

### Analyses of miRNA-mRNA Targets

We applied the miRNA-target tool ENCORI to predict the miRNA of DEGs (24). There were nine databases about miRNA-mRNA prediction. miRNAs predicted in at least two databases were selected as the potential target miRNAs of hubgenes. Then, the Cytoscape software was employed to assess the regulatory networks of the miRNA-mRNA pairs.

### Immune Cell Infiltration

The immune cell components in adipose tissue were analyzed *via* CIBERSORT (25).

### Prediction of Drugs Targeting DEGs

The Drug–Gene Interaction Database (DGIdb, www.dgidb.org) is a web resource that organizes and presents gene druggability information and drug-gene interactions from databases, articles, and web resources (26). Herein, we used DGIdb (version 3.0.2) to predict the potential drugs that target key DEGs confirmed *via* network module analysis. The following parameters were used: Preset filter, Food, and Drug Administration approved; advanced filters, source databases: all; gene categories, all; and interaction types, all. The interaction network was constructed using Cytoscape.

### Statistics

Comparison of the clinical characteristics was achieved using the 2-tailed Student’s t-test for continuous variables or the χ2 test for dichotomous variables. A p-value is less than 0.05 denoted significance. Adipocyte size and positive cell numbers were compared *via* a 2-tailed Student’s t-test. For the qRT-PCR experiment, expression values relative to β-actin were compared using 2-way ANOVA and Tukey’s multiple comparisons test.

## Results

### Clinical Characteristics of Subjects

**Table 2** outlines the clinical characteristics of 31 subjects. The mean age of the CAD group and control group were 66.06 ± 7.94 years and 60.93 ± 12.03 years, respectively (P = 0.165). Except for a trend towards more female patients in the control group (17.6% *vs* 42.9%, P = 0.124) and diabetes history in the CAD group (7/17 *vs* 2/14, P=0.101), we reported no difference in age, gender, BMI, comorbid conditions, and clinical biochemical characteristics. As such, our study groups were well matched. The clinical features of 9 cases who underwent RNA sequencing are outlined in **Supplemental Table 1**. Discriminant multivariate analysis demonstrated that the two groups were well matched.

**Table 2:** Clinical characteristics of the subjects.

Parameters	CAD (n=17)	NCAD (n=14)	p value
Sex (male/female)	14/3	8/6	0.124
Age (years), mean±SD	66.06 ± 7.94	60.93 ±12.03	0.165
BMI (kg/m2), mean±SD	24.7 ± 3.48	25.69 ± 4.26	0.489
Hypertension (Yes/No)	10/7	7/7	0.623
Diabetes (Yes/No)	7/10	2/12	0.101
Stroke (Yes/No)	4/13	1/13	0.217
Smoking (Yes/No)	4/13	1/13	0.217
Total-cholesterol (mM), mean±SD	4.15 ± 1.19	4.55 ± 1.12	0.350
Triglycerides (mM), mean±SD	1.90 ± 1.36	1.50 ± 0.58	0.293
HDL-cholesterol (mM), mean±SD	1.10 ± 0.18	1.31 ± 0.42	0.200
LDL-cholesterol (mM), mean±SD	2.62 ± 1.24	2.45 ± 0.76	0.725
ESR (mm/h), mean±SD	13.44 ± 12.39	14.31 ± 6.77	0.835
CRP (mg/L), mean±SD	10.33 ± 14.55	6.49 ± 5.64	0.368
WBC (10^9/L), mean±SD	6.71 ± 1.39	6.41 ± 2.31	0.662
N (%), mean±SD	66.06 ± 10.09	66.53 ± 13.42	0.913
Hb (g/L), mean±SD	135.88 ± 14.73	132.57 ± 21.58	0.617
ALT (mM), mean±SD	29.61 ± 22.70	29.10 ± 15.10	0.944
AST (mM), mean±SD	25.91 ± 12.02	24.91 ±9.77	0.805
BUN (mM), mean±SD	6.36 ± 1.64	5.84 ± 2.13	0.446
CR (mM), mean±SD	79.04 ± 15.47	81.99 ± 30.72	0.748
eGFR, mean±SD	88.69 ± 14.90	84.67 ± 23.50	0.579
UA (mM), mean±SD	364.46 ± 93.92	386.91 ± 137.50	0.594

### Depot-Specific Transcriptomic Profiles of PAT in CAD and Control Groups

RNA-seq was performed in 9 patients (CAD=5, Control=4), we first sought to identify depot-specific gene signatures in a pairwise manner. Using threshold criteria of fold change (FC) greater than or equal to 2 or less than or equal to –2 and p-value less than or equal to 0.05, 147 differentially expressed genes (DEGs) (104 upregulated and 43 downregulated) were identified in the CAD group relative to the control group (i.e., CAD vs. control groups) (**Figures 1B, C**). In addition, the top significantly changing genes were IL6, FOSB, CSF3, IGLC7, and PPP1R14C; these genes were associated with inflammation, nuclear transcription, cell differentiation, immunity, and neuronal activity.

### Function and Pathway Enrichment Analyses

To explore the relative functions and pathways of DEGs in CAD *vs*. control groups, we performed Gene Ontology (GO) and Kyoto Encyclopedia of Genes and Genomes (KEGG) analysis on those DEGs. The DEGs were significantly enriched in regulation of acute inflammatory response, regulation of complement activation, and complement activation classical pathway associated biological process (BP) terms, circulating immunoglobulin complex, immunoglobulin complex, and external side of plasma membrane associated component cell (CC) terms, antigen binding, RNA polymerase II-specific immunoglobulin receptor binding, and DNA-binding transcription activator activity associated molecule function (MF) terms (**Figure 1E**). KEGG pathway analysis demonstrated the enrichment of DEGs in cytokine-cytokine receptor interaction, IL-17 signaling pathway, and COVID-19 (**Figure 1D**).

### PPI Network Construction and Module Analysis of DEGs

**Figure 2:**
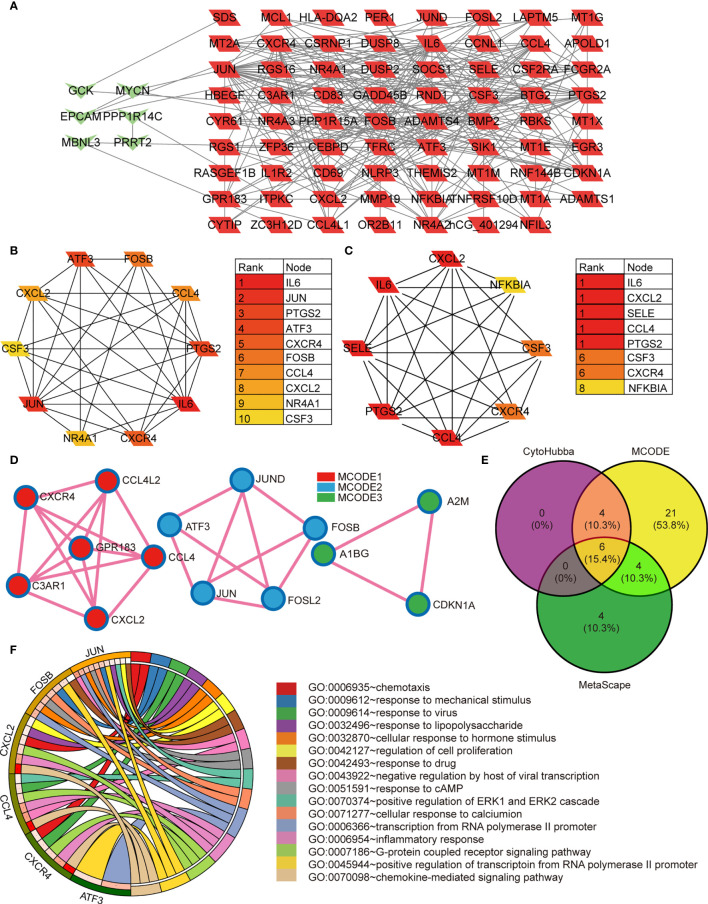
**(A)** The protein-protein interaction network constructed based on the DEGs. The green heart represents the downregulated genes, the red parallelogram represents the upregulated genes. **(B–D)** The hubgenes predicted by cytohubba, Mcode, and metascape Mcode, respectively. **(E)** Overlap of hubgenes identified *via* the three methods; **(F)** The Pathway analysis results showing the six enriched hubgenes.

We systematically analyzed the biological functions of the obtained DEGs between the two groups using a PPI network of DEGs constructed *via* the STRING database and visualized using Cytoscape. The PPI network comprised 77 nodes (proteins) and 271 edges (interactions; **Figure 2A**). The 77 nodes included 71 up-regulated genes and 6 down-regulated genes. According to the topology score, Interleukin-6 (IL-6), Jun proto-oncogene (JUN), and prostaglandin-endoperoxide synthase 2 (PTGS2) activating transcription factor 3 (ATF3), chemokine (C-X-C motif) receptor 4 (CXCR4) were the top five genes (**Figure 2B**). Additionally, five MCODE modules were identified from the PPI network *via* cytoscape_CMODE; however, one submodule with a score >5 was extracted from the PPI network, which comprised 8 nodes and 26 edges (**Figure 2C**). In this module, IL-6, chemokine (C-X-C motif) ligand 2 (CXCL2), selectin E (SELE), C-C motif chemokine ligand 4 (CCL4), PTGS2 were the top five genes (**Figure 2C**). Three MCODE modules were extracted through Metascape analysis (**Figure 2D**).

### Identification and Analysis of Significant Genes

**Figure 3:**
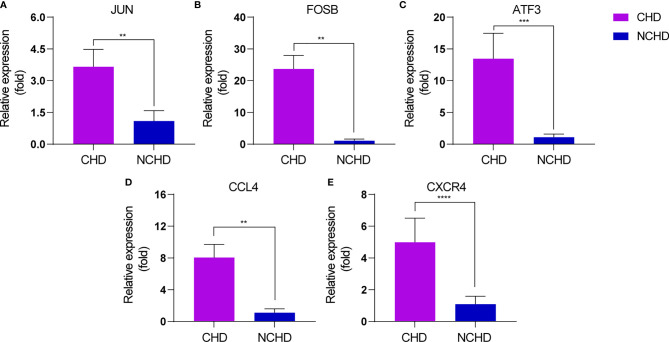
**(A–E)** RT-PCR verification of the expression levels of 5 genes (JUN, FOSB, ATF3, CCL4, CXCR4). The 2−ΔΔCT method was used to analyze the relative expression levels of various genes. * means 0.05 < p<0.1; ** means p < 0.05; *** means p < 0.01; **** means p < 0.001.

To reveal the most important hub gene, we filtered these genes using the VENN diagram. The VENN diagram revealed six significant common genes, including JUN, ATF3, CXCR4, FOSB, CCL4, and CXCL2 (**Figure 2E**). The functions of the six significant genes are summarized in **Table 3**. We shifted our focus to explore the potential function of these six key genes. Pathway analysis demonstrated that the six genes were mainly enriched in the chemokine-mediated signaling pathway, inflammatory response, transcription from RNA polymerase II promoter, G-protein coupled receptor signaling pathway, and positive regulation of transcription from RNA polymerase II promoter (**Figure 2F**). RT-qPCR analysis revealed that the relative expression levels of JUN (**Figure 3A**), FOSB (**Figure 3B**), ATF3 (**Figure 3C**), CCL4 (**Figure 3D**), and CXCR4 (**Figure 3E**) were significantly higher in the CAD group adipose tissue than in control group.

**Table 3:** The enrichment results of GO and KEGG of hubgenes.

Category	Term	P value
GO	chemokine activity	0.000104
	chemokine receptor binding	0.000213
	cytokine activity	0.002367
	cytokine receptor binding	0.003134
	G protein-coupled receptor binding	0.003652
KEGG	IL-17 signaling pathway	1.49E-05
	Viral protein interaction with cytokine and cytokine receptor	1.79E-05
	Chemokine signaling pathway	0.000126
	Cocaine addiction	0.000354
	Cytokine-cytokine receptor interaction	0.000452

### miRNA-mRNA Interaction Network

**Figure 4:**
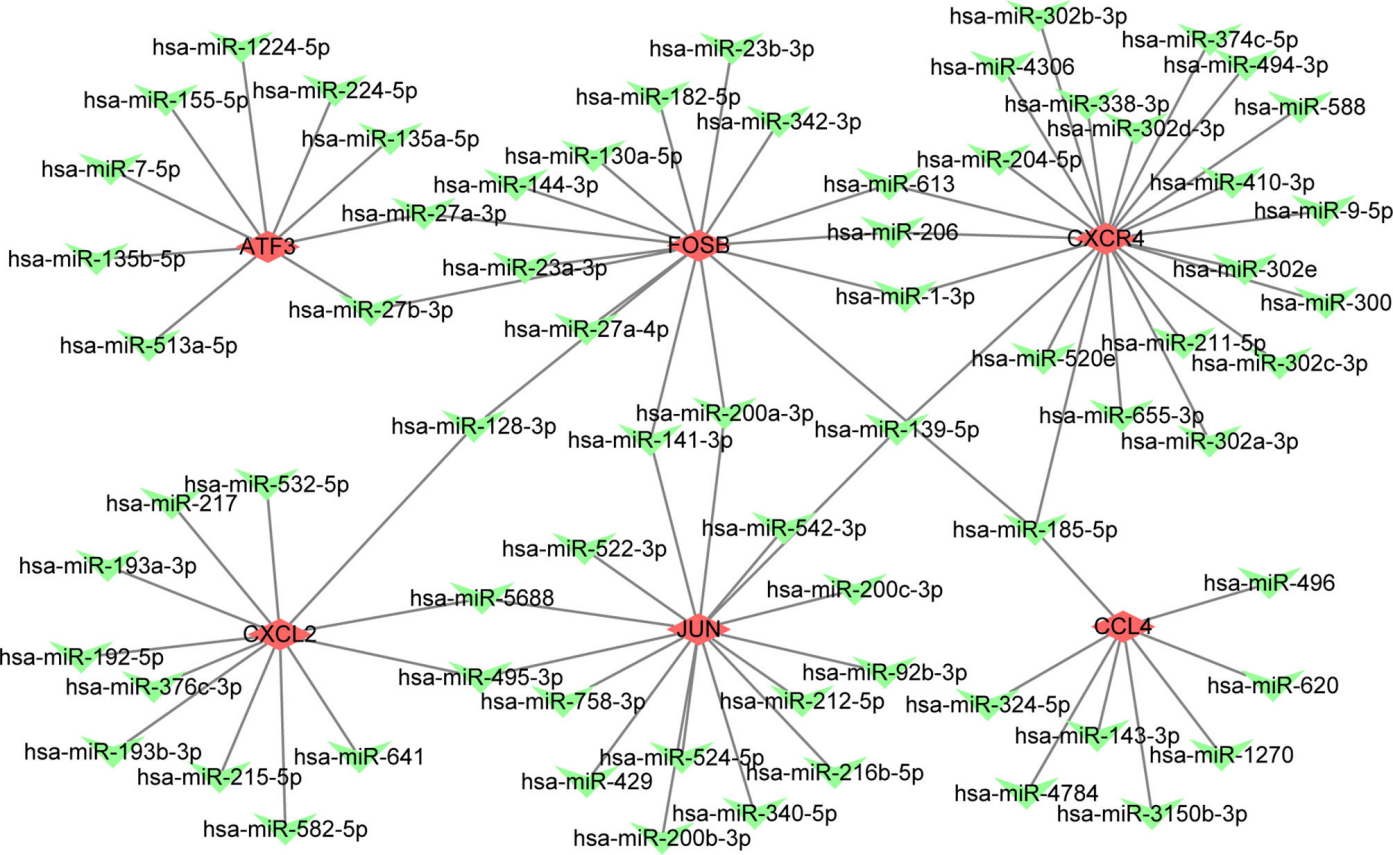
The constructed mRNA-miRNA network. The green heart represents the downregulated genes, the red diamond represents the hubgenes.

We explored the effect of PAT on CAD progression and its potential gene regulation mechanism. miRNA-target gene interaction pairs of reverse association were predicted *via* ENCORI according to the hub genes identified previously. Considering the identified miRNA-mRNA pairs, we compared the interaction network containing 70 miRNA-mRNA pairs and visualized them *via* the Cytoscape software. Through comparison of the targets of hub genes, we found CXCR4 to be a potential target of 23 miRNAs, including hsa-miR-185-5p. Also, FUN and FOSB were the potential targets of 15 miRNAs. The miRNA-gene regulation network is illustrated in **Table 4** and **Figure 4**, respectively.

**Table 4:** The predicted miRNA about 6 hubgenes.

Gene symbol	Count	miRNA		
JUN	15	hsa-miR-92b-3p	hsa-miR-524-5p	hsa-miR-200c-3p
		hsa-miR-758-3p	hsa-miR-522-3p	hsa-miR-200b-3p
		hsa-miR-5688	hsa-miR-495-3p	hsa-miR-200a-3p
		hsa-miR-542-3p	hsa-miR-429	hsa-miR-141-3p
		hsa-miR-216b-5p	hsa-miR-340-5p	hsa-miR-139-5p
ATF3	9	hsa-miR-1224-5p	hsa-miR-135b-5p	hsa-miR-224-5p
		hsa-miR-135a-5p	hsa-miR-155-5p	hsa-miR-27a-3p
		hsa-miR-27b-3p	hsa-miR-513a-5p	hsa-miR-7-5p
CXCR4	24	hsa-miR-9-5p	hsa-miR-494-3p	hsa-miR-302d-3p
		hsa-miR-655-3p	hsa-miR-4306	hsa-miR-302c-3p
		hsa-miR-613	hsa-miR-410-3p	hsa-miR-302b-3p
		hsa-miR-588	hsa-miR-374c-5p	hsa-miR-302a-3p
		hsa-miR-520e	hsa-miR-338-3p	hsa-miR-300
		hsa-miR-204-5p	hsa-miR-302e	hsa-miR-211-5p
		hsa-miR-185-5p	hsa-miR-1-3p	hsa-miR-206
		hsa-miR-139-5p	hsa-miR-27a-4p
FOSB	15	hsa-miR-613	hsa-miR-27a-3p	hsa-miR-200a-3p
		hsa-miR-342-3p	hsa-miR-23b-3p	hsa-miR-185-5p
		hsa-miR-27b-3p	hsa-miR-23a-3p	hsa-miR-182-5p
		hsa-miR-141-3p	hsa-miR-130a-5p	hsa-miR-144-3p
		hsa-miR-1-3p	hsa-miR-128-3p	hsa-miR-212-5p
CCL4	8	hsa-miR-620	hsa-miR-4784	hsa-miR-3150b-3p
		hsa-miR-496	hsa-miR-324-5p	hsa-miR-185-5p
		hsa-miR-143-3p	hsa-miR-1270	
CXCL2	12	hsa-miR-641	hsa-miR-532-5p	hsa-miR-217
		hsa-miR-582-5p	hsa-miR-495-3p	hsa-miR-215-5p
		hsa-miR-5688	hsa-miR-376c-3p	hsa-miR-193b-3p
		hsa-miR-193a-3p	hsa-miR-192-5p	hsa-miR-128-3p

### Macrophages Are the Major Immune Infiltrating Cell Subset

**Figure 5:**
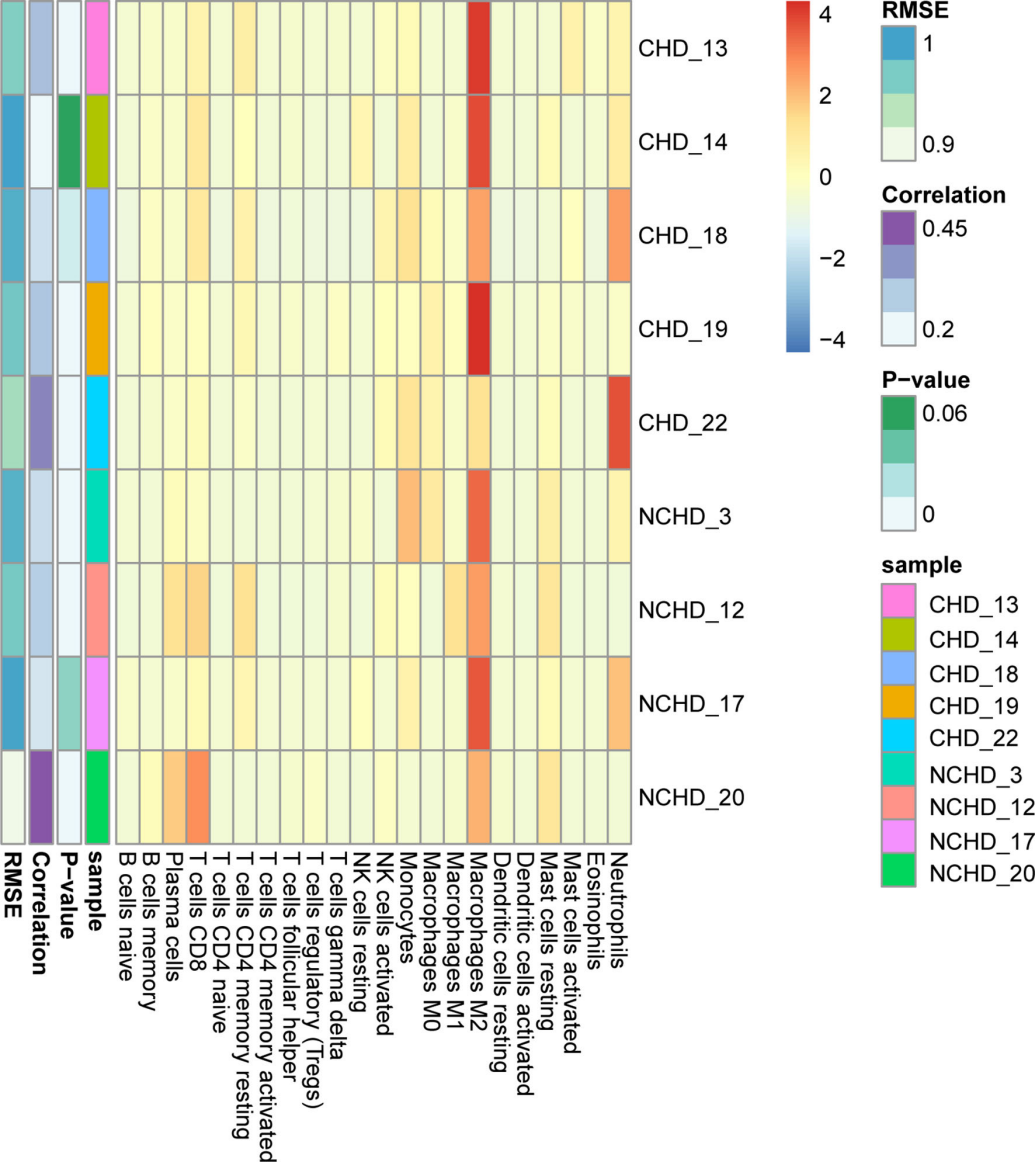
Distinction of infiltrating immune cell subpopulations and levels between CHD/NCHD groups.

**Figure 6:**
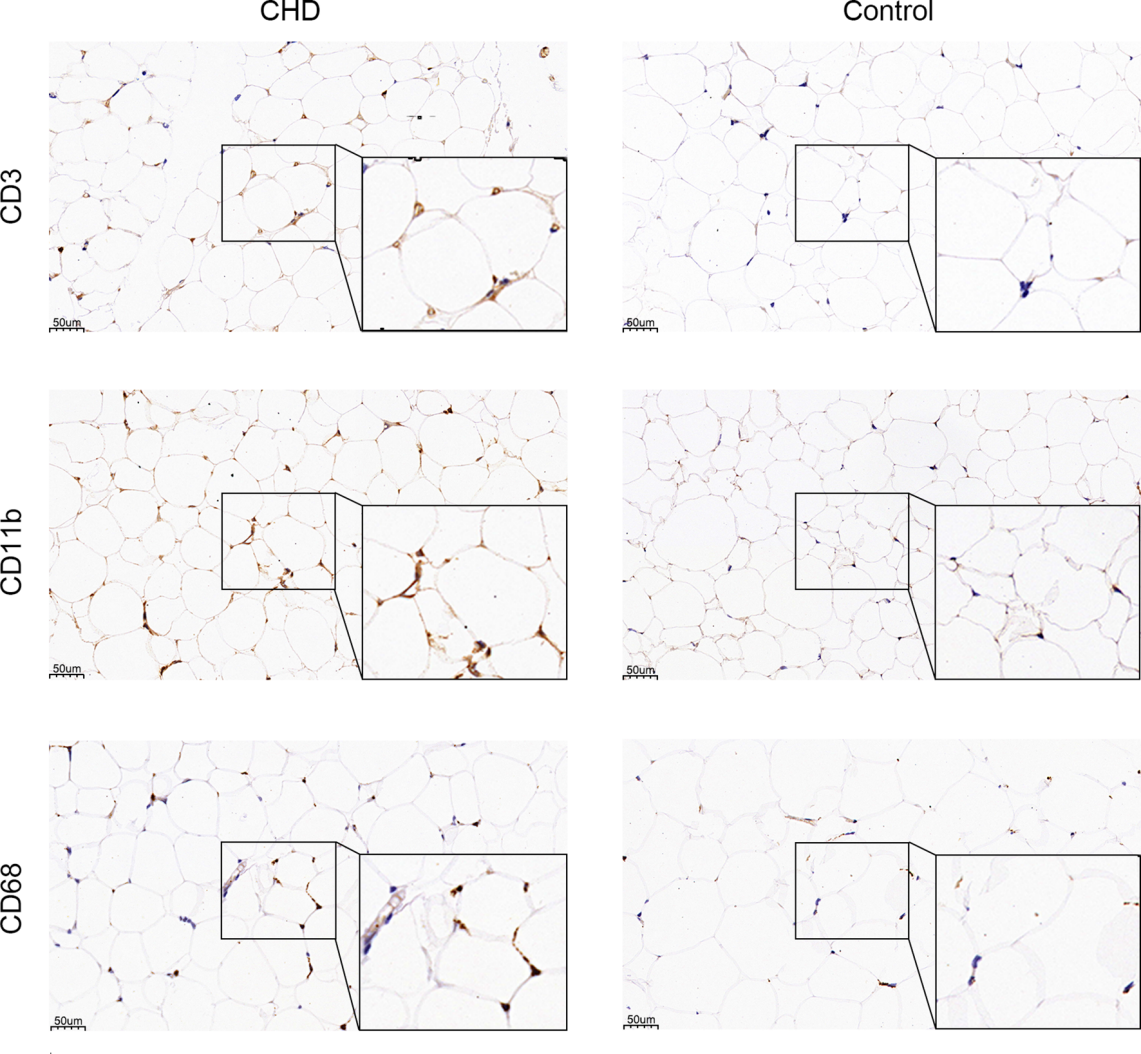
IHC staining of CD68, CD11b, and CD3. CD68, CD11b, and CD3 protein are stained in brown.

GO and KEGG enrichment analysis has shown inflammatory response is the major procession in PAT of CAD patients. However, the component of immune cells in the pericardial adipose deposit is unknown. We employed CIBERSORT, a bioinformatics tool used to infer immune cell composition from RNA-seq datasets, to compute the relative frequency of 22 infiltrating immune cell subsets in the 9 cases. Results revealed that monocytes and macrophages were the most common immune cell subsets with mean fractions of 0.098 and 0.325, respectively (**Figure 5**). Further, we verified the inflammatory phenotype of human PAT from both CAD and control patients through immunohistochemistry analysis of markers of T cell (CD3), macrophage (CD68), and its M1 subtype (CD11b). We found that PAT from CAD patients had significantly more T cells (CD3+) and macrophages (CD68+) than that of control patients (**Figure 6**). Semi-quantification analysis revealed that the IOD/Area of macrophages and T-cells in PAT of CAD patients was significantly different (**Table 5**). These findings further proved that PAT exerts a potential inflammatory effect in the regulation of CAD.

**Table 5:** The semi-quantification analysis of infiltrating immune cell.

Cell type	CHD	Contral
T cells (IOD/Area)	0.093	0.075
Macrophages (IOD/Area)	0.337	0.177

### Drug Predictable Results

**Figure 7:**
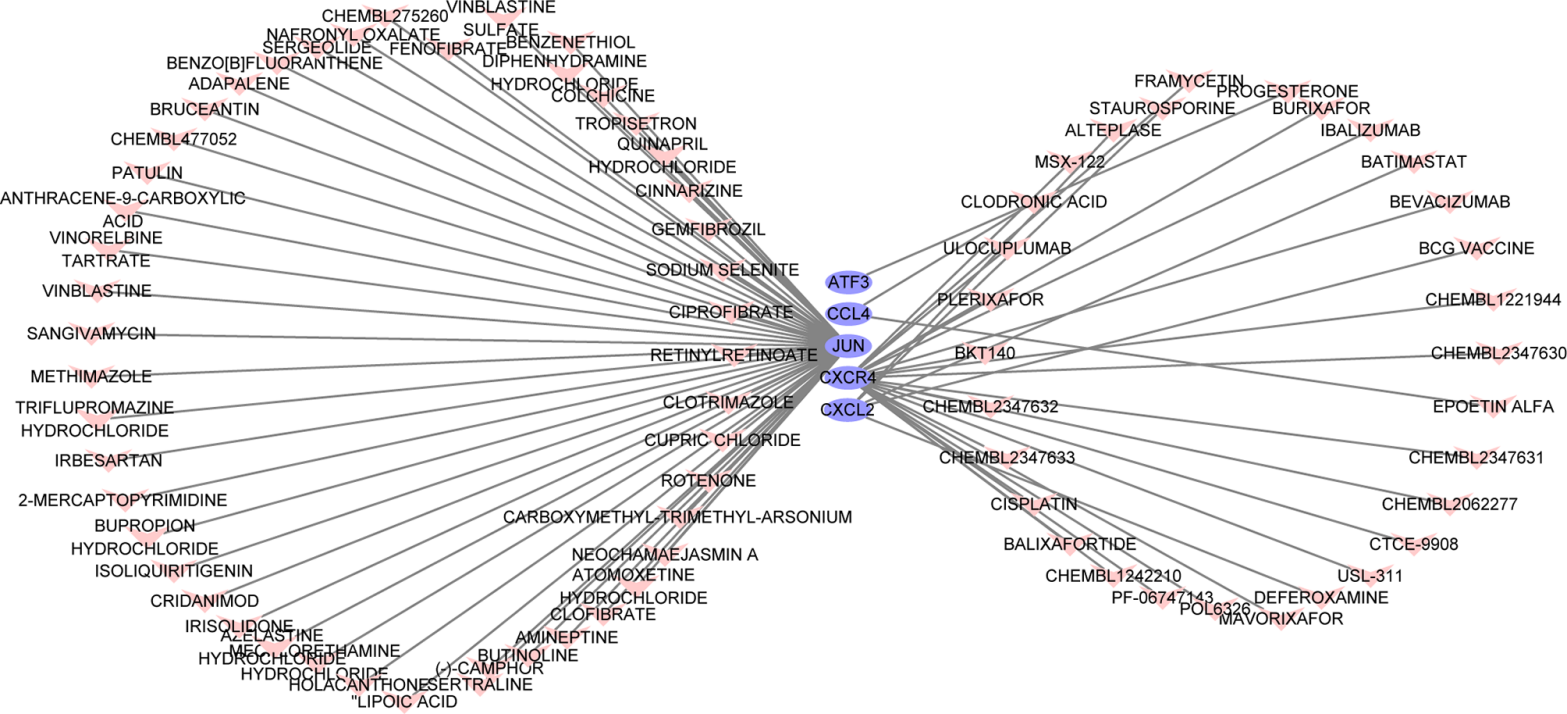
The constructed mRNA-drug network. The purple oval represents the genes, and the light red heart represents the drugs.

The search for potential drugs that can improve the function of PAT to intervene in the disease process of CAD propelled us to analyze the six key genes for potential drugs *via* DGIdb. Finally, five genes were considered as druggable genes, including JUN, CXCL2, and CXCR4; also we obtained 77 possible drugs. An interaction network including 82 nodes and 77 edges was constructed based on the five druggable genes (**Figure 7**).

## Discussion

In this study, we have sequenced the transcriptome of PAT from five patients with CAD and four patients without CAD and analyzed the DEGs between two groups. A total of 147 DEGs (104 up-regulated genes and 43 down-regulated genes) were identified. We subjected the DEGs to GO functional and KEGG pathway enrichment analyses *via* Clusterprofile package, and obtained 40 significant GO terms and 55 significant KEGG pathways. GO term also demonstrated the interaction of these genes with immune and inflammatory cells, and we, therefore, employed the Cibersort online tool (https://cibersort.stanford.edu/) to analyze 22 types of immune cells infiltration. Based on the results, monocytes and macrophages were the most common immune cell subsets. Further immunohistochemical staining demonstrated that more macrophages were present in PAT from CAD patients, and the proportion of M1 subtype cells was higher. These findings imply that PAT inflammation potentially plays a crucial role in CAD progression.

Moreover, GO and KEGG pathway analysis results revealed the significant enrichment of DEGs in cytokine-cytokine receptor interaction, IL-17 signaling pathway, and COVID-19, all of which are inflammatory processes. Compelling evidence implicates the IL-17 signaling pathway in the pathogenesis of many inflammatory-driven conditions, including atherosclerosis (27–30). A study found that IL-17 maintained plaque stability through induction of proliferation of SMCs and collagen content in atherosclerotic plaques (29). Also, IL-17 had been revealed to potentially lower the expression of vascular cell adhesion molecule (VCAM)-1 in endothelial cells and prevent monocyte adherence and block T cell infiltration into plaques (31). Other reports show that IL-17 can induce the release of chemokines, including CXCL1, CXCL2, CXCL8, CXCL10. Subsequently, these chemokines can recruit neutrophils and monocytes to the atherosclerotic lesion (32, 33). Furthermore, IL-17 can stimulate macrophages to produce inflammatory cytokines, such as IL-6, TNF-a, and IL-1b (34–36), and induce apoptosis of vascular endothelial cells through activation of caspase-3 and caspase-9. These observations affirm that the IL-17 signaling pathway mediates atherosclerosis (37). We also found that other signaling pathways such as NF-kappa B signaling pathway, TNF signaling pathway, JAK-STAT signaling pathway, and Toll-like receptor signaling pathway were enriched in PAT of CAD patients. Of note, some proteins in these pathways can interplay with each other, thereby increasing the severity of inflammation and apoptosis (38). These results imply that the management of inflammation in CAD may require nonspecific inflammatory inhibitors.

The PPI network-integrated three modules in the analysis of six significant and reproducible genes (JUN, ATF3, CXCR4, FOSB, CCL4, CXCL2), and revealed differential expression between the CAD and control groups. Three core genes (CXCR4, CXCL2, and CCL4) were markedly enriched in the chemokine signaling pathway, whereas the others (Jun, FosB, and ATF3) were transcription factors. Chemokines are cytokines that mediate cell chemotaxis and stagnation as they bind to their respective cell surface receptors (39). Chemokines induce the aggregation of inflammatory cells to the inflammatory site, which is the main factor that trigger vascular inflammatory injury (39, 40). Previous reports indicate that CCL4 is highly expressed in atherosclerotic patients (41), and it potentially exerts a crucial role in the progression of atherosclerotic plaque (42–44). In addition, inhibition of CCL4 can stabilize atherosclerotic plaques by decreasing the expression of MMPs, inflammatory cell infiltration, circulation of inflammatory factors, and regulation of blood lipid levels (43). The C-X-C motif chemokine receptor 4 (CXCR4) is a cytokine receptor and mediates various biological processes (45). The ligand of CXCR4 is C-X-C motif chemokine ligand 12 (CXCL12) (46). CXCL12 and CXCR4 coordinatively play a pivotal role in atherosclerosis and arterial injury (47, 48). CXCR4 expression in leukocytes is closely associated with the vulnerability of atherosclerosis plaque (49). A recent study found that CXCR4-positive macrophages accumulated in tissue samples of human carotid plaques and, CXCR4 was expressed in both smooth muscle cell progenitors and endothelial progenitor cells in atherosclerotic plaque progression (46). The study by Puca AA showed that upregulation of CXCR4 decreases the development of atherogenic process. This is because it can skew macrophages to acquire an M2-resolving phenotype, maintains maintain arterial integrity, preserves preserve endothelial cell integrity, and restore the normal contractile SMC phenotype (50, 51). In contrast to CXCR4, CCL4 inhibition reduced the adhesiveness of coronary endothelial cells, which is an early sign of atherogenesis (43). Herein, we found that CCL4, CXCR4 derived from PAT was highly expressed at the mRNA level in CAD patients. More exploration of CCL4 and CXCR4 secreted by PAT would enhance the understanding of the pathophysiological mechanism of atherosclerosis and facilitate its utilization as a biomarker and intervention target for atherosclerosis.

Activator protein-1 (AP-1) is an important class of nuclear transcription factors in the body. It is a homodimer or heterodimer composed of JUN, FOS, ATF, and MAF protein families which exert biological effects (52). AP-1 is largely associated with cell proliferation, differentiation, apoptosis, and inflammation (53–55). Previously, the AP-1 cascade was found to potentially drive additional leukocyte recruitment to the developing atherosclerotic matrix (56). AP-1 could also promote the development of atherosclerosis by inducing endothelial cell death, the proliferation and migration of vascular smooth muscle cells (57, 58). In this study, we observed a significant increase in mRNA expression of AP-1 factors JUN, FOSB, and ATF3 from PAT in CAD patients. These results imply that PAT-derived AP-1 has potential effects on the progression of the coronary artery through cell proliferation, inflammation, and extracellular matrix remodeling.

Based on bioinformatics, we constructed the miRNA-mRNA network of core genes through the ENCORI database to further elucidate the regulatory mechanism of core genes. Among the predicted miRNAs, only miRNA-185-5p bound to three of the six core genes (FOSB, CCL4, and CXCR4). Previous studies had confirmed that miRNA-185-5p is widely involved in the proliferation, metastasis, and inflammation of various tumors (59–61). However, its regulatory role in inflammation and the progression of CAD remains elusive.

Through the prediction of the Drug-Gene Interaction database, we revealed some drugs that potentially interact with the core genes. Some drugs were used in the clinic but not to manage CAD. For instance, colchicine, an anti-inflammatory alkaloid, has been mainly used in gout diseases (62); it exerts inhibitory effects on leukocyte chemotaxis and adhesion, microtubule assembly, and reduces the production of inflammatory mediators (63, 64). Because of its anti-inflammatory properties, the feasibility of the value of colchicine in CAD had been investigated in several clinical trials. Most clinical trials suggest that colchicine is beneficial in patients with CAD or myocardial infarction (65–67). However, some studies revealed that colchicine could reduce major cardiovascular events (68), however, the overall mortality did not decrease. As such, colchicine may increase the mortality of non-cardiovascular events and limit its application in CAD (69), which warrants more large-scale clinical trials. Fenofibrate is a broad-spectrum lipid-lowering drug, which acts on PPAR to potentially reduce cholesterol and triglyceride (70, 71). Elsewhere, a study found that fibrates have potential anti-inflammatory effects, protect endothelial cell function, and improve insulin resistance (72). However, these drugs are not recommended for the secondary prevention of CAD. In clinical trials such as HHS, VA-HIT, and BIP, fibrates have proved to exhibit significant benefits in subgroups of patients, including those with insulin resistance or metabolic syndrome (73–75). Recent studies have further outlined that fibrates can reduce the risk of cardiovascular events and the level of uric acid in diabetic patients with dyslipidemia (76, 77). Collectively, fibrates may have great clinical prospects in some specific groups of patients with CAD.

In summary, this work has allowed for the identification of the transcriptome characteristic shift in PAT from humans with or without CAD and revealed the upregulation in inflammatory processes of PAT in CAD patients. The crucial genes, pathways, and drug target genes closely associated with CAD have been revealed through bioinformatics analyses. Particularly, the critical genes are potential biomarkers and therapeutic targets in CAD. Also, colchicine and fenofibrate are the most promising drugs for coronary artery disease.

## Limitations of the Study

The primary limitation of the research is that we did not analyze samples from healthy subjects; this could have been more interesting for the PAT physiology study. Besides, the analyzed samples from older patients with some comorbidity who came in for various cardiac surgeries rather than no comorbidity. We also acknowledge that we had a rather small RNA-Seq sample size, which could not allow us to analyze samples grouped by obesity, age, and gender. This may have contributed to the CAD-associated transcriptomic shifts.

## Data Availability Statement

The datasets presented in this study can be found in GEO (Gene Expression Ominbus), a public functional genomics data repository. The accession number(s) in the GEO can be found below: GSE179397.

## Ethics Statement

The studies involving human participants were reviewed and approved by the ethics committee of Huadong Hospital Affiliated to Fudan University, Shanghai, China. The patients/participants provided their written informed consent to participate in this study.

## Author Contributions

ML and LQ have contributed equally to this work and share first authorship. All authors contributed to the article and approved the submitted version.

## Funding

This work was supported by the Science and Technology Commission of Shanghai Municipality (20140900600), National Natural Science Foundation of China (81770420, 91949126, 81970378), Shanghai Municipal Key Clinical Specialty (shslczdzk02801), National Key Research and Development Program of China (2020YFC2009001), and Center of geratic coronary artery disease.

## Conflict of Interest

The authors declare that the research was conducted in the absence of any commercial or financial relationships that could be construed as a potential conflict of interest.

## Publisher’s Note

All claims expressed in this article are solely those of the authors and do not necessarily represent those of their affiliated organizations, or those of the publisher, the editors and the reviewers. Any product that may be evaluated in this article, or claim that may be made by its manufacturer, is not guaranteed or endorsed by the publisher.
