# Supplementary material for: Association of Pericardiac Adipose Tissue With Coronary Artery Disease
Source: Front Endocrinol (Lausanne). 2021 Sep 6;12:724859. doi: 10.3389/fendo.2021.724859 (PMC8451419; doi:10.3389/fendo.2021.724859)
Supplement: Supplementary file 1 [file Table_1.docx]

| Supplemental Table 1: Clinical characteristics of the selected subjects. | | | |
| --- | --- | --- | --- |
| Parameters | CAD(n=5) | NCAD(n=4) | p value |
| Sex(male/female) | 5/0 | 3/1 | 0.292 |
| Age(years),mean±SD | 63.80±8.70 | 62.50±17.74 | 0.889 |
| BMI(kg/m2) ,mean±SD | 25.88±3.41 | 25.64±1.22 | 0.901 |
| Hypertension(Yes/No) | 4/1 | 1/3 | 0.125 |
| Diabetes(Yes/No) | 3/2 | 1/3 | 0.356 |
| Stroke(Yes/No) | 3/2 | 1/3 | 0.356 |
| Smoking(Yes/No) | 3/2 | 0/4 | 0.068 |
| Total-cholesterol(mM) ,mean±SD | 4.35±0.77 | 4.39±1.56 | 0.963 |
| Triglycerides(mM) ,mean±SD | 1.58±0.82 | 1.51±0.61 | 0.889 |
| HDL-cholesterol(mM) ,mean±SD | 1.15±0.28 | 1.30±0.63 | 0.673 |
| LDL-cholesterol(mM) ,mean±SD | 2.70±0.70 | 2.64±1.06 | 0.931 |
| ESR(mm/h) ,mean±SD | 10.40±5.50 | 12.75±3.20 | 0.477 |
| CRP(mg/L),mean±SD | 8.81±2.02 | 6.87±2.69 | 0.254 |
| WBC(10^9/L) ,mean±SD | 6.92±1.51 | 6.79±2.16 | 0.915 |
| N(%),mean±SD | 70.24±10.46 | 71.08±13.06 | 0.918 |
| Hb(g/L) ,mean±SD | 135.00±14.63 | 130.00±30.14 | 0.715 |
| ALT(mM) ,mean±SD | 23.28±11.61 | 17.25±6.89 | 0.393 |
| AST(mM) ,mean±SD | 24.50±10.26 | 19.03±4.86 | 0.363 |
| BUN(mM) ,mean±SD | 5.62±1.78 | 6.38±1.32 | 0.504 |
| CR(mM) ,mean±SD | 85.28±8.22 | 94.83±29.73 | 0.509 |
| eGFR,mean±SD | 84.39±9.20 | 76.25±29.31 | 0.571 |
| UA(mM) ,mean±SD | 399.80±65.80 | 419.48±163.03 | 0.347 |
